# Positive Charges Put the Brakes on Ribosomes

**DOI:** 10.1371/journal.pbio.1001509

**Published:** 2013-03-12

**Authors:** Roland G. Roberts

**Affiliations:** Public Library of Science, Cambridge, United Kingdom

**Figure 1:**
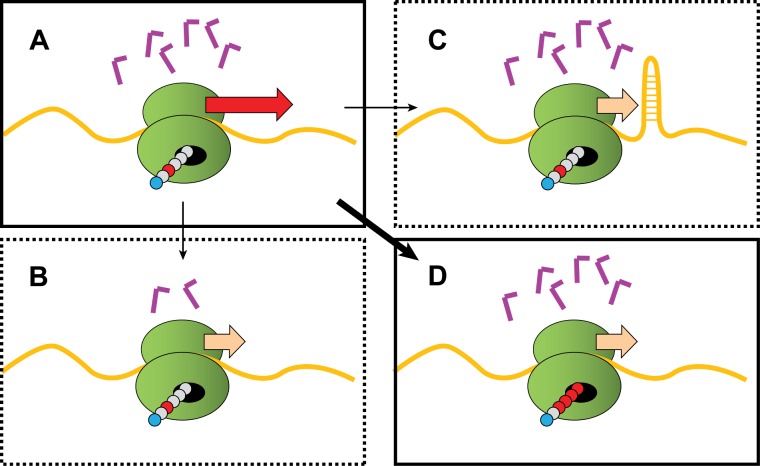
What slows ribosomes down? The freely translating ribosome (A) was thought to be slowed by codons that need rare tRNAs (B) or by mRNA secondary structure (C). Charneski and Hurst, however, show that the dominant braking factor is the presence of positively charged amino acids (red beads) in the exit tunnel (D).

The ribosome is at the core of cellular life. It's the incredible machine that trundles along gene transcripts (mRNAs), translating them three letters at a time into the proteins that run most of the workings of the cell. The translation process requires little L-shaped adapters called tRNAs—one end of these recognizes a specific three-letter word (codon) on the mRNA, while the other end carries the corresponding amino acid. The ribosome then glues the amino acids together and shoves the growing protein chain out through an exit tunnel in the back of the ribosome (see Figure 1A).

But a question that has occupied many minds over the years is whether the ribosome moves at a more-or-less constant rate along mRNA molecules, or whether its speed is influenced by factors beyond its control. This interest comes from several lines of endeavor. One comprises evolutionary biologists who want to understand the natural optimization of protein translation and the forces that shape it. Another is biotechnologists who aspire to optimize translation speed artificially to maximize protein productivity. The speed of translation is also known to affect the folding and localization of the final protein product—issues of broader physiological impact.

Studies on single transcripts seem to show that translation speed is indeed highly variable, and the orthodoxy is that a major determinant of ribosome speed is the abundance of the tRNA molecule needed to interpret each codon. Not all tRNAs are equal, so a codon needing a rare tRNA might slow translation because the ribosome has to wait longer to encounter that tRNA (Figure 1B), and vice versa. Another presumed major player is mRNA secondary structure— if the ribosome needs to plow through a tangled segment of transcript, then it might slow down as energy is expended to unravel the obstacle (Figure 1C).

What's changed in the last few years is that rather than relying on data from individual transcripts, the newly developed technique of ribosome profiling gives us a snapshot of where the ribosomes are across all the transcripts in the cell. The resulting “ribosomal occupancy” data can be analyzed to infer a high-resolution pan-transcriptomic map of ribosome speeds that has the potential to tell us exactly what might be slowing ribosomes down.

So what *is* the answer? In this issue of *PLOS Biology*, Catherine Charneski and Laurence Hurst show that the prime determinant of ribosome speed isn't actually a direct feature of the mRNA itself, but a feature of the emerging protein in the exit tunnel (Figure 1D). Using a previously published dataset, the authors are able to exclude codon/tRNA abundance (Figure 1B) and mRNA secondary structure (Figure 1C) as the most important factors. Ruling out these and all other explanations they could think of, and excluding confounding factors, the authors find out what really seems to matter: if a ribosome has just translated one or more positively charged amino acids (lysine, arginine and, to a lesser extent, histidine), then it slows down. The proposed explanation for this surprisingly simple outcome is that the positive charges interact electrostatically with the negatively charged lining of the ribosomal exit tunnel, gumming it up. The longer the run of positive charges, the greater the effect.

The finding that most variation in translation speed could be explained (like romance on the London Underground) largely by opposites attracting in a tunnel challenges the prevailing complex view based on codon usage and mRNA secondary structure; this may well prove controversial, but it brings a provocative clarity to an issue of broad significance. As a tantalizing postscript, the authors also wonder whether the polyA tails that decorate the ends of mRNAs (and which would encode the positively charged amino acid lysine if translated) might've evolved as a sand-trap for runaway ribosomes.

**Charneski CA, Hurst LD (2013) Positively Charged Residues Are the Major Determinants of Ribosomal Velocity. doi:10.1371/journal.pbio.1001508**

